# Cytochrome c Oxidase Activity as a Metabolic Regulator in Pancreatic Beta-Cells

**DOI:** 10.3390/cells11060929

**Published:** 2022-03-08

**Authors:** Genya Aharon-Hananel, Leonor Romero-Afrima, Ann Saada, Carmit Mantzur, Itamar Raz, Sarah Weksler-Zangen

**Affiliations:** 1 The Hadassah Diabetes Center, Hadassah Medical Center, Jerusalem 9112102, Israel; genya.ah@gmail.com (G.A.-H.); leonoromero@gmail.com (L.R.-A.); carman03@gmail.com (C.M.); ntv502@netvision.net.il (I.R.); 2 Division of Endocrinology, Diabetes and Metabolism, The Chaim Sheba Medical Center, Tel Hashomer, Ramat-Gan 5266202, Israel; 3 Sackler School of Medicine, Tel Aviv University, Tel Aviv 6997801, Israel; 4 The Department of Genetics, Hadassah Medical Center, Jerusalem 9112102, Israel; annsr@hadassah.org.il; 5 Faculty of Medicine Hebrew, University of Jerusalem, Jerusalem 9112102, Israel; 6 The Liver Research Laboratory, Hadassah Medical Center, Jerusalem 9112102, Israel

**Keywords:** cytochrome c oxidase, glucose stimulated insulin secretion, pancreatic β-cells, blood glucose levels

## Abstract

Pancreatic β-cells couple glucose-stimulated insulin secretion (GSIS) with oxidative phosphorylation via cytochrome c oxidase (COX), a mitochondrial respiratory-chain enzyme. The Cohen diabetic-sensitive (CDs) rats exhibit hyperglycemia when fed a diabetogenic diet but maintain normoglycemia on a regular diet. We have previously reported a decreased COX activity in CDs rats and explored its relevance for type 2 diabetes (T2D). In this study, we investigated the relation between COX activity in islets, peripheral-blood mononuclear cells (PBMCs), and GSIS during diabetes development in CDs rats fed a diabetogenic diet for 4, 11, 20, and 30 days and during reversion to normoglycemia in hyperglycemic CDs rats fed a reversion diet for 7, 11, and 20 days. An oral glucose-tolerance test was performed at different periods of the diets measuring blood glucose and insulin concentrations. COX activity was determined in islets and PBMCs isolated from rats at the different periods of the diets. We demonstrated a progressive reduction in COX activity in CDs-islets that correlated positively with the decreasing GSIS (R^2^ = 0.9691, *p* < 0.001) and inversely with the elevation in blood glucose levels (R^2^ = 0.8396, *p* < 0.001). Hyperglycemia was initiated when islet COX activity decreased below 46%. The reversion diet restored >46% of the islet COX activity and GSIS while re-establishing normoglycemia. Interestingly, COX activity in PBMCs correlated significantly with islet COX activity (R^2^ = 0.8944, *p* < 0.001). Our data support islet COX activity as a major metabolic regulator of β-cells function. The correlation between COX activity in PBMCs and islets may serve as a noninvasive biomarker to monitor β-cell dysfunction in diabetes.

## 1. Introduction

Pancreatic β-cell dysfunction and the ensuing impaired glucose-stimulated insulin secretion (GSIS) are essential for the progression from pre-diabetes to diabetes [1,2,3]. Glucose influx triggers insulin secretion through a coordinated process requiring mitochondrial Adenosine triphosphate (ATP) generation up to the metabolic threshold needed for robust insulin release and glucose control [3,4,5]. The rise in the ATP/ADP (Adenosine diphosphate) ratio is coordinated by oxidative phosphorylation (OxPhos) carried out by five mitochondrial respiratory chain (MRC) multimeric-enzyme complexes [3,4,5]. Cytochrome c oxidase (COX, Complex-IV), the terminal hetero-oligomeric heme–copper oxidase, transfers electrons from reduced cytochrome c to molecular oxygen [5,6,7].

Mitochondrial OxPhos is crucial for GSIS, and as such, it could play a central role in the pathophysiology of type 2 diabetes (T2D). In accordance with this, islets from T2D donors demonstrated fused or fragmented mitochondria and a selective impairment in GSIS, while insulin secretion in response to non-fuel secretagogues was maintained [8,9,10,11,12]. The reduced expression of a set of OxPhos genes was demonstrated in islets of animal models of diabetes and patients with T2D [13,14], and impairment in the assembly of the MRC super-complex was observed in animal models of diabetes [15] as well as in the rectus abdominis muscle of obese individuals with T2D [16]. Additionally, mitochondrial dysfunction was demonstrated in platelets of both diabetic rats and T2D patients [17], suggesting that peripheral-blood mononuclear cells (PBMCs) may serve as a biomarker to observe the mitochondrial changes during T2D [18].

We previously demonstrated a markedly reduced COX activity in islets of hyperglycemic Cohen Diabetic Sensitive (CDs) rats fed a diabetogenic diet for 30 days [19,20,21,22]. The purpose of this study was to assess the longitudinal changes in islets’ COX activity during the progression to hyperglycemia and reversion of normoglycemia while establishing the minimal COX activity required while maintaining normoglycemia. We further evaluated whether COX activity in PBMCs may serve as a noninvasive biomarker for detecting mitochondrial dysfunction in the islets.

## 2. Materials, Methods and Research Design

### 2.1. Animals and Diets

Rats: CDs and Control (CDr that do not develop diabetes on the diabetogenic diet) rats are bred and maintained in the animal facility at the Hebrew University School of Medicine, Jerusalem. Diets: Rats were fed ad libitum one of the following three diets according to the study protocol (Table 1). (1) Regular diet (RD) (Teklad, 2018, Harlan Laboratories, Placentia, CA, USA), composed of 54% carbohydrate (ground whole wheat, alfalfa, and bran); 21% protein (skimmed milk powder); 6% fat; 5% salts, vitamins, and trace elements including an adequate copper content (15 ppm); 7% humidity; and 7% ash. (2) Diabetogenic diet; CDs rats maintaining normoglycemia on RD [22] were switched to a custom-prepared diabetogenic diet, high sucrose low copper diet (HSD), containing 72% sucrose; 18% vitamin-free casein; 5% salt-mixture no. II USP (MP Biomedicals, Solon, OH, USA); 4.5% butter; and 0.5% corn oil, vitamins and low copper (0.9 ppm) [22]. (3) Reversion diet, a custom prepared chow consisting of HSD supplemented with adequate amounts (16 ppm) of copper [20]. The Institutional Animal Experiments Committee (IACUC) approved these animal studies, approval Code: MD-18-15403-3.

### 2.2. Study Design

Eight week old male CDs and Control rats fed RD (time point “0”) were switched to the “diabetes progression” protocol, the diabetogenic diet feeding for 4, 11, 20, or 30 days (*n* = 5–8 rats for each period on the diabetogenic diet, Table 1). Hyperglycemic CDs rats fed 30 days with the diabetogenic diet were switched to a “diabetes reversion” protocol in which they were fed a reversion diet for 4, 7, 11, or 20 days (*n* = 5–8 rats for each period on the reversion diet, Table 1). At the end of the periods on the diets, an oral glucose tolerance test (OGTT) was performed to assess the glucose metabolism measuring the blood glucose and insulin concentration as described previously [20,21]. Islets and PBMCs were isolated from CDs and Control rats for COX activity measurements as described previously [19,20]. Hyperglycemia in CDs rats was defined as 2 h post-OGTT blood glucose levels > 11 and normoglycemia as <7.8 (1 mmol/L) [20,21,22].

### 2.3. Oral Glucose Tolerance (OGTT) Testing

Overnight fasted rats fed according to the progression and reversion protocols underwent an OGTT. The blood glucose and insulin concentration were measured following overnight fasting (0) and 30, 60, 90, and 120 min after gavage administration of glucose (350 mg glucose/100 g b.wt). Blood glucose concentrations were measured using a glucometer (EliteR, Bayer, Leverkusen, Germany). Serum insulin concentrations were measured using an ELISA assay (Mercodia AB, Uppsala, Sweden).

### 2.4. Islets Isolation

Following OGTT, islets were isolated as previously described [19]. Briefly, the pancreas was distended-digested with collagenase-P (Roche Molecular Biochemicals, Indianapolis, IN, USA). Islets were hand-picked, counted, and rapidly frozen in liquid nitrogen. Homogenates of 200–300 islets of comparable size were used for enzymatic-activity assays.

### 2.5. PBMCs Isolation

PBMCs were obtained from 5 ml of whole blood drawn in EDTA by gradient centrifugation using Lymhoprep (Axis-Shield, Dundee, UK) according to the manufacturer’s instructions. Pelleted isolated PBMCs were stored at −70 °C until the enzymatic activity assay [23].

### 2.6. Assessment of the Mitochondrial Enzymatic Activity

COX’s activity and the ubiquitous mitochondrial matrix enzyme citrate-synthase (CS), used as a mitochondrial marker enzyme, were determined by standard spectrophotometric methods [24]. Briefly, COX activity was measured by following the oxidation of reduced cytochrome c at 550 nm. CS was measured in the presence of acetyl-CoA and oxaloacetate by monitoring the liberation of CoASH coupled to 5′5-dithiobis (2-nitrobenzoic) acid at 412 nm. Enzymatic activities are expressed as a ratio normalized to CS activity [19].

### 2.7. Data Presentation and Statistical Analysis

Data were analyzed using two-way ANOVAs with Bonferroni’s post-test to compare multiple columns using SigmaStat (Jandel Corporation, San Rafael, CA, USA). COX activity was calculated as a ratio of COX/CS, both measured in the same sample. The glucose levels and insulin-secretion area under the curve (AUC) were calculated using the trapezoidal integration of the 120 min OGTT. Insulin-secretion is presented as the “insulinogenic-index”, the insulin/glucose ratio calculated at each time point in the OGTT. The correlation of islets and PBMCs COX activity with the glucose levels and insulinogenic index was analyzed using the Pearson correlation. Data are presented as means ± SEM from duplicates or triplicates from at least three independent experiments for each data point.

## 3. Results

### 3.1. OGTT of CDs and Control Rats Fed According to the Progression or Reversion Protocols

**Figure 1:**
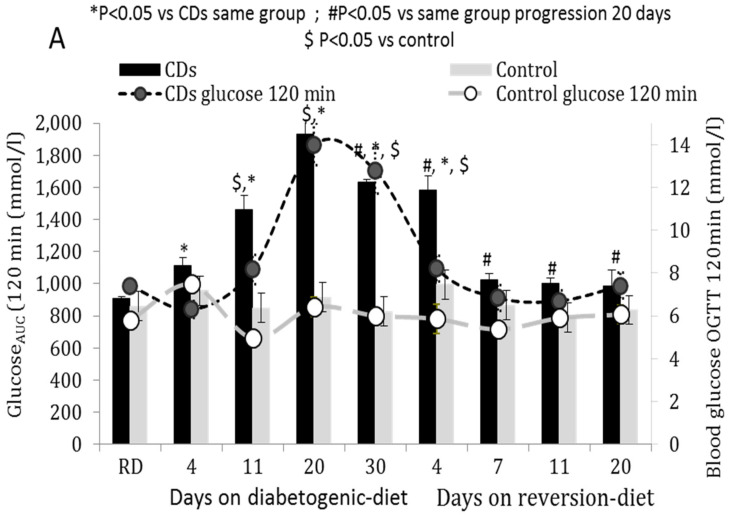

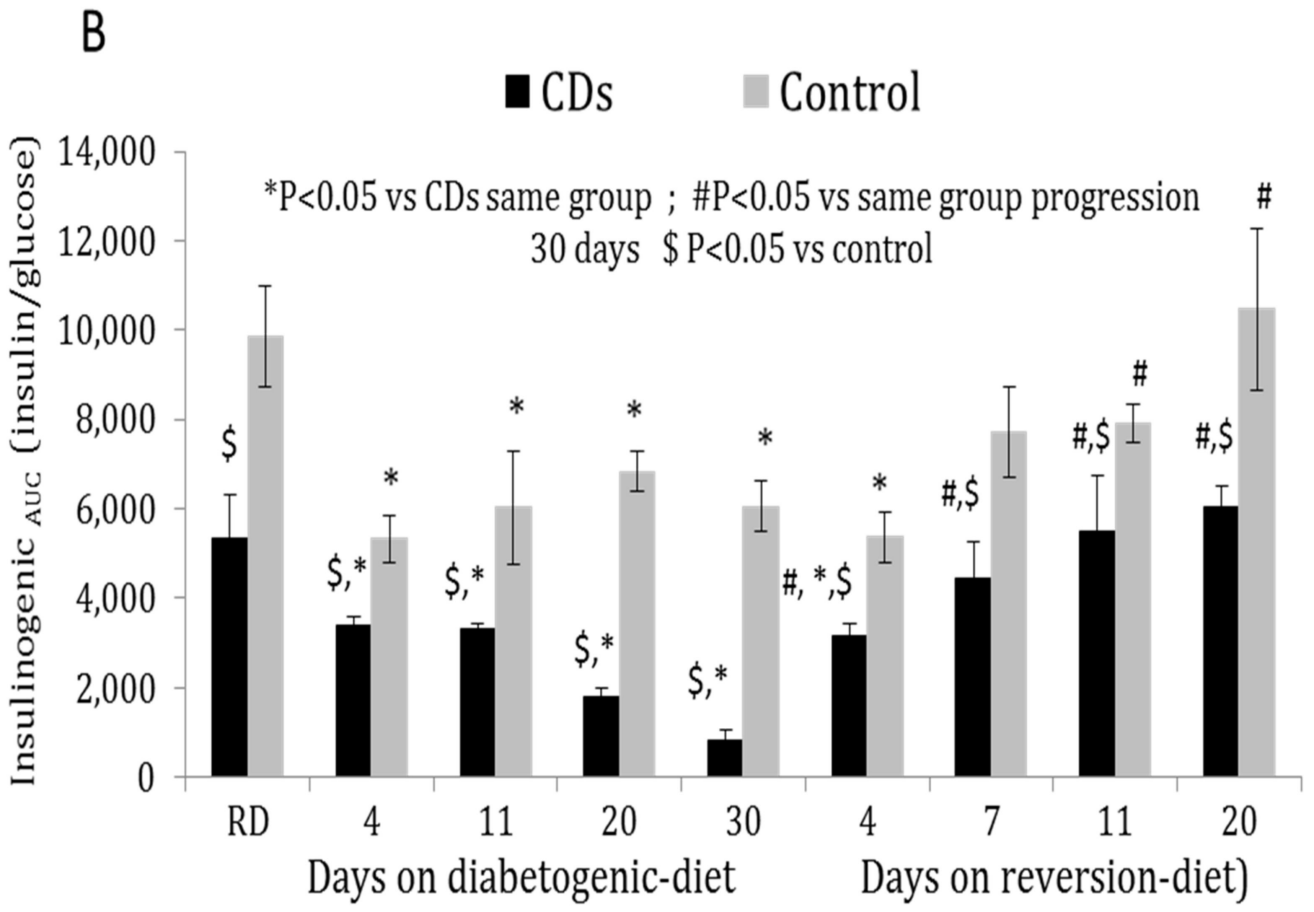
(**A**,**B**) Area under the curve (AUC) of (**A**) blood glucose and (**B**) plasma insulin (calculated as insulin/glucose, “insulinogenic-index”) determined before (overnight fast, 0) and after (30, 60, 90, 120 min) glucose administration (350 mg/100 g b.wt) in CDs and Control rats fed RD and a diabetogenic diet for 4, 11, 20 and 30 days and a reversion diet for 4, 7, 11, and 20 days. The light (CDs) and dark (CDr) grey curves represent the two-hour post-OGTT glucose and insulin levels. Data are means ± SEM of 5–8 independent experiments, * *p* < 0.05 RD, # *p* < 0.05 vs. rats on diabetogenic diet, $ *p* < 0.05 vs. control, *n* = 5–8 rats/feeding period.

CDs rats fed with the diabetogenic diet exhibited a gradual time-dependent increase in the glucose area under the OGTT curve (AUC_glucose_, Figure 1A) paralleled by a time-dependent decrease in AUC_insulin_ calculated as the insulinogenic index (insulin/glucose) (Figure 1B). A short-term exposure (four days) to the diabetogenic diet significantly reduced AUC_insulin_ (*p* < 0.01 vs. CDs RD, Figure 1B), yet normoglycemia was maintained (Figure 1A light-grey curve representing two h post-OGTT glucose levels of <7.8 mmol). An impaired glucose tolerance (glucose levels > 7.8 mmol/L two h post-OGTT) was first observed on the 11th day of the diabetogenic diet, and overt hyperglycemia (>11.1 mmol/L) was observed on the 20th day (Figure 1A) (*p* < 0.001 vs. CDs RD) paralleled with a marked decrease in GSIS (Figure 1B). The gradual reduction in blood glucose levels towards normoglycemia observed when fed the reversion diet was paralleled with an increase in AUC_insulin_ (Figure 1A,B). Four days on the reversion protocol significantly reduced glucose levels, yet a normal glucose tolerance was fully restored only on day 7 (*p* < 0.05) and normoglycemia was reached only when >50% GSIS (AUCinsulin) was restored (*p* < 0.01 vs. CDs RD) (Figure 1A,B).

Control rats maintained normoglycemia at all time points of the diabetogenic and reversion diets. GSIS significantly decreased but was held above 50% for all periods of the diabetogenic diet, increasing back to baseline at 20 days of the reversion diet (Figure 1A,B).

### 3.2. COX Activity in Isolated Pancreatic Islets and Peripheral PBMCs

**Figure 2:**
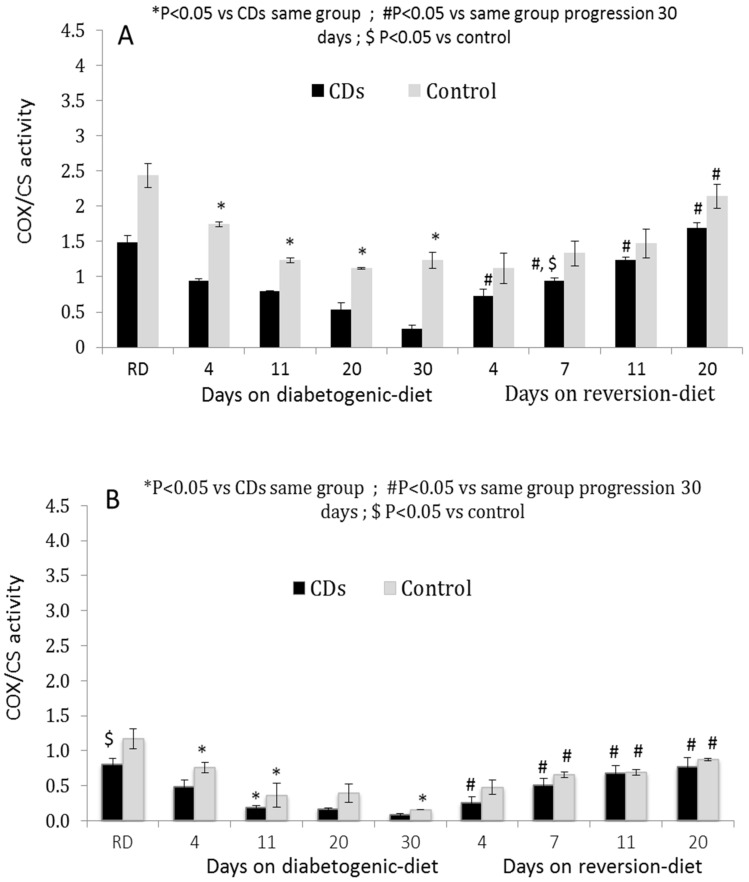
(**A**,**B**) COX-activity in (**A**) islets and (**B**) PBMCs isolated from CDs and control rats fed RD and a diabetogenic diet for 4, 11, 20, and 30 days and the reversion diet for 4, 7, 11, and 20 days, (*n* = 5–8 rats/feeding period). Data are means ± SEM of COX/CS activity, * *p* < 0.05 RD, # *p* < 0.05 vs. diabetogenic diet; $ *p* < 0.05 vs. control.

Islets and PBMCs isolated from CDs rats maintained on the diabetogenic diet exhibited a gradual time-dependent decrease in COX activity paralleled by a reduction in GSIS (Figure 2A,B). The COX activity decreased significantly following a short-term (four days) exposure to the diabetogemic diet (*p* < 0.01, Figure 2). On the 20th day, islets COX activity decreased to less than half, and after 30 days only a residual of <20% remained (*p* < 0.01 vs. normoglycemic CDs RD islets). The COX activity recovered partially to 62% of baseline by day seven on the reversion diet and was fully restored to the level of CDs RD after 20 days. COX activity in the islets of Control rats was higher than that of the CDs islets at all time points of the diabetogenic and reversion diets. The islet COX activity gradually declined also in islets of the Control rats on the diabetogenic diet maintaining at least 46% activity (of baseline) and was significantly higher than COX activity in islets of CDs rats at all periods on the diabetogenic and reversion diets (Figure 2A). The COX activity in the PBMCs followed a similar pattern to that of the islets (Figure 2B).

This suggests that a minimal level of ≥46% islet COX activity is required to sustain >50% GSIS and normoglycemia. In accordance with this, Control rats maintain ≥46% islet COX activity during all periods on the diabetogenic and normoglycemia while CDs rats exhibit less than 46% islet COX activity and develop hyperglycemia.

### 3.3. Correlation of COX Activity in Islets and PBMCs with Post-OGTT Glucose Levels and GSIS

**Figure 3:**
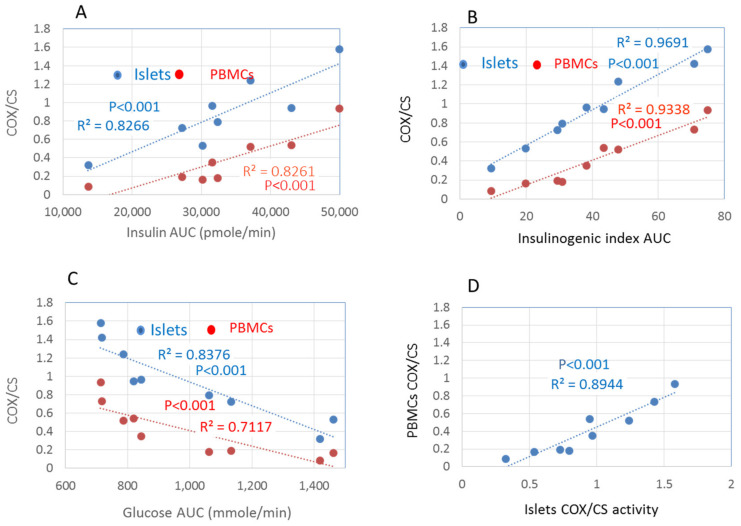
(**A**,**D**) Pearson correlation between islets COX/CS activity ratio (open circle), PBMCs COX/CS activity ratio (black circle), and (**A**) the insulinogenic index_AUC_ (insulin/glucose) or (**B**) AUC glucose of CDs rats fed diabetogenic and reversion-diets. (**C**) Pearson correlation between islets vs. PBMCs COX activity (**D**). Data are means ± SEM of 5–8 independent experiments.

The CDs islet COX activity was positively correlated with AUC GSIS presented as the insulin and insulinogenic index (R^2^ = 0.8266 and 0.9691, respectively, *p* < 0.001) (Figure 3A,B) and inversely correlated with blood glucose levels (R^2^ = −0.8376, *p* < 0.001, Figure 3C). The COX activity in peripheral PBMCs significantly correlated with the COX activity in islets isolated from the same rats at matching periods of diabetogenic and reversion diets (R^2^ = 0.8944, *p* < 0.001, Figure 3D), supporting the notion that PBMCs’ COX activity may be used as a noninvasive biomarker to monitor β-cell function.

## 4. Discussion

T2D is preceded by years of prediabetes, during which the elevation of blood glucose exerts deleterious effects on β-cell mitochondria and impairs insulin secretion [2,4,5,8,11,12,25,26,27]. On the other hand, disrupting the mitochondrial oxidative metabolism blocks GSIS [1,2,3]. Thus, the role of β-cell mitochondrial dysfunction as a primary defect in T2D is still unresolved. This study provides evidence for a critical role of defective islet COX activity as a preliminary event leading to diabetes in CD rats. We examined the relationship between reduced islet COX activity and decreased GSIS in a longitudinally designed study measuring islet COX activity, GSIS, and blood glucose levels. We found that the reduced GSIS was almost perfectly correlated with the decreasing islet COX activity and increased blood glucose levels (Figure 3B), implicating a significant role for islet COX activity as a modulator of GSIS. We also found that COX activity in PBMCs was highly correlated with islet COX activity during both the progression and reversion of diabetes, suggesting that PBMCs could reflect islet COX activity and may serve as a non-invasive biomarker (Figure 3C).

The role of mitochondrial dysfunction in the pathophysiology of T2D has long been debated. An abnormal mitochondrial morphology and reduced GSIS have been found in β cells from postmortem T2D patients [8,9,10]. Impairments in OxPhos [16,28,29] and diminished mitochondrial activity have been demonstrated in diabetes patients [2,4,11,12,27,30,31,32,33,34,35], while mutations in the mitochondrial genome such as the mtDNA 3243 mutation were shown to be associated with diabetes [36,37,38,39,40]. More specifically, OxPhos genes were differentially expressed, and DNA methylation was found in these genes in islets from patients with T2D compared with nondiabetic donors [13,14]. Impairments in OxPhos activity [16,28,29] and differentially expressed OxPhos genes were found in islets from patients with T2D compared with nondiabetic donors [13,14] as well as in animal models of diabetes [15,41], supporting a pivotal role of the mitochondria OxPhos in β-cell dysfunction. However, data regarding COX dysfunction and its role in pancreatic islets and β-cell function are limited.

**Figure 4:**
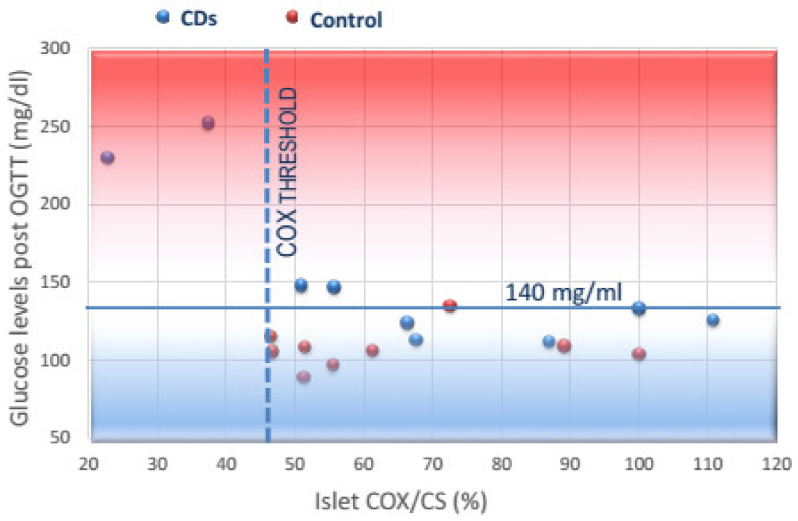
A proposed scheme describing the relationship between islet COX activity and blood glucose levels in CDs and Control rats according to the different times spent on the diabetogenic and reversion diets. Each circle represents a time period in the diabetogenic/reversion diets. Normoglycemia (blue zone, 2 h post-OGTT blood glucose levels < 140 mg/dL) was observed in rats that had islet COX activity above the minimal COX activity threshold (46% of baseline), while hyperglycemia (red zone 2 h post-OGTT blood glucose levels > 200 mg/dL) was observed when COX activity decreased below the threshold. This “islet COX activity threshold” seems to be mandatory to sustain normoglycemia in this rat model.

In the current study, we detected that a minimal level of islet COX activity was required to sustain long-term normoglycemia in CDs rats. The idea of a “threshold” controlling GSIS had been suggested two decades ago for glucokinase activity, catalyzing the first and rate-limiting step of glycolysis [42]. In mitochondrial diseases exhibiting a dysfunctional OxPhos, mitochondrial respiration remained nearly maximal until COX activity was decreased below a >50% threshold [43,44]. In accordance with this, in the current study, when the islet COX activity is reduced below 46% of baseline, hyperglycemia develops, as schematically presented in Figure 4. This scheme illustrates that when COX activity decreased by ≥30% (to 66–70% of baseline COX activity) in islets isolated from CDs rats fed four days on the diabetogenic diet, normal blood glucose levels sustained. When COX activity drops furthermore in islets isolated from CDs rats fed 11 days the diabetogenic diet, blood glucose levels increased to 150 mg/dl inducing impaired glucose tolerance. Overt hyperglycemia developed when the islet COX activity dropped below the threshold of ≥46 of baseline. This may suggest that a minimal islet COX activity is required to sustain normoglycemia in CDs rats, defining a “metabolic islet COX activity threshold” of 46% activity of baseline required to sustain long-term normoglycemia. Moreover, the observation that the decreases in the islet COX activity may be reinstated above the critical threshold, reestablishing normal blood glucose levels, supports islet COX activity as a targetable regulator for improving glucose homeostasis.

This observation is in line with the “metabolic threshold” attributed to other enzymes controlling GSIS [42,45]. However, to the best of our knowledge, this is the first time that an islet COX activity threshold was defined in relation to glucose homeostasis.

Different thresholds were described to explain the variability of energy metabolism in mitochondrial diseases caused by genetic defects in mitochondrial or nuclear DNA encoding complexes of the electron transport chain (ETC) [44]. The studies showed that, in most cases, a phenotypic manifestation of the genetic defect occurs only when a threshold level is exceeded, and this phenomenon was named the “phenotypic threshold effect”. Subsequently, several authors demonstrated that it was possible to considerably inhibit the activity of a respiratory chain complex, up to a critical value, without affecting the rate of mitochondrial respiration or ATP synthesis. This phenomenon was called the “biochemical threshold effect”. A “biochemical threshold effect” was described in different organs and for other ETC complexes in isolated rat mitochondria and permeabilized human cells [44,46] but has not yet been demonstrated in pancreatic islets.

The inability of CDs mitochondria to maintain COX activity above the critical islet COX activity threshold could be due to the failure to activate compensatory mechanisms in the islets. It is well-established that the activation of AMP-activated protein kinase (AMPK) is physiologically induced by conditions of a shortage of ATP [47]. Reduced AMPK was observed in islets of T2D and was associated with reduced insulin secretion [48]. Activating the AMPK/PPARgamma Coactivator 1 alpha (Pgc1α) axis was shown to correct OxPhos defects in vivo [47,48,49]. In the current study, we did not include data evaluating the AMPK/PGC1α axis. However, in preliminary studies, we observed a decreased expression of genes involved in mitochondrial biogenesis in islets of hyperglycemic CDs rats (data not shown).

Another possibility is that the CDs rats harbor mutations in the OxPhos-related nuclear gene, which could affect COX assembly, resulting in an inborn 50% reduction in COX activity. The manifestation of diabetes will then occur when shifted to a harmful environment of the diabetogenic diet, inducing an additional decrease in the islet COX activity below the critical threshold resulting in hyperglycemia.

As human pancreatic islets are not accessible for diagnostic purposes, we searched for an accessible, less invasive biomarker to predict islets COX activity. PBMCs were selected based on earlier studies showing a reduced mitochondrial complex-I activity in PBMCs of T2D patients. An impaired platelet mitochondrial function was found in diabetic rats and patients [17,18]. We found COX activity in PBMCs to correlate significantly with COX activity in islets, thus suggesting that they might serve as a biomarker for islet COX activity. The assessment of the metabolic function in human blood for treatment and disease diagnosis is an innovative and vital area of translational research [50,51]. PBMCs circulate permanently through the body and are exposed to the pancreatic environment. They are likely capable of reflecting the characteristic pathologies of internal tissues in response to nutritional interventions. This opens the possibility that these circulating cells could sense metabolic stress in patients and serve as biomarkers of mitochondrial dysfunction in human pathologies such as diabetes.

In conclusion, our findings link reduced mitochondrial COX activity in islets, insulin secretion, and glucose homeostasis, suggesting that islet COX activity could be a significant metabolic sensor in pancreatic β-cells. We also identified a novel islet COX activity threshold required to sustain normoglycemia and identified a possible noninvasive biomarker that could be used to monitor β-cell failure. Identifying and characterizing the mechanisms by which reduced COX activity leads to β-cell failure and finding predictors of β-cell failure are essential steps in elucidating the pathogenesis of T2D and identifying potential targets for therapeutic interventions [29,52].

## Figures and Tables

**Table 1:** Diets and study protocol.

	Period on the Diet (days)	# of Rats
Diets		
Regular diet (RD)	30	40
Diabetogenic High Sucrose diet (HSD)	4, 11, 20, 30	5–8 rats/period on the diet
Reversion diet	4, 7, 11, 20	5–8 rats/period on the diet

## Data Availability

The data are available from the corresponding authors upon reasonable request.
